# Comparison of an AI-driven planning tool and manual radiographic measurements in total knee arthroplasty

**DOI:** 10.1016/j.csbj.2025.04.009

**Published:** 2025-04-12

**Authors:** Marie Theres Heller, Guenther Maderbacher, Marie Farina Schuster, Lina Forchhammer, Markus Scharf, Tobias Renkawitz, Stefano Pagano

**Affiliations:** Department of Orthopedic Surgery, University of Regensburg, Asklepios Klinikum, Bad Abbach, Germany

**Keywords:** Total Knee Arthroplasty, Artificial Intelligence, Automated Planning, Radiographic Measurement, Intraclass Correlation Coefficient, Measurement Efficiency

## Abstract

**Background:**

Accurate preoperative planning in total knee arthroplasty (TKA) is essential. Traditional manual radiographic planning can be time-consuming and potentially prone to inaccuracies. This study investigates the performance of an AI-based radiographic planning tool in comparison with manual measurements in patients undergoing total knee arthroplasty, using a retrospective observational design to assess reliability and efficiency.

**Methods:**

We retrospectively compared the Autoplan tool integrated within the mediCAD software (mediCAD Hectec GmbH, Altdorf, Germany), routinely implemented in our institutional workflow, to manual measurements performed by two orthopedic specialists on pre- and postoperative radiographs of 100 patients who underwent elective TKA. The following parameters were measured: leg length, mechanical axis deviation (MAD), mechanical lateral proximal femoral angle (mLPFA), anatomical mechanical angle (AMA), mechanical lateral distal femoral angle (mLDFA), joint line convergence angle (JLCA), mechanical medial proximal tibial angle (mMPTA), and mechanical tibiofemoral angle (mTFA).

Intraclass correlation coefficients (ICCs) were calculated to assess measurement reliability, and the time required for each method was recorded.

**Results:**

The Autoplan tool demonstrated high reliability (ICC > 0.90) compared with manual measurements for linear parameters (e.g., leg length and MAD). However, the angular measurements of mLPFA, JLCA, and AMA exhibited poor reliability (ICC < 0.50) among all raters. The Autoplan tool significantly reduced the time required for measurements compared to manual measurements, with a mean time saving of 44.3 seconds per case (95 % CI: 43.5–45.1 seconds, *p* < 0.001).

**Conclusion:**

AI-assisted tools like the Autoplan tool in mediCAD offer substantial time savings and demonstrate reliable measurements for certain linear parameters in preoperative TKA planning. However, the observed low reliability in some measurements, even amongst experienced human raters, suggests inherent challenges in the radiographic assessment of angular parameters. Further development is needed to improve the accuracy of automated angular measurements, and to address the inherent variability in their assessment.

## Highlights

• AI tool shows high reliability for linear TKA measures.
• Autoplan drastically cuts time for TKA measurement vs. manual.
• Angular TKA measurements by AI currently show lower reliability.
• Study compares AI and expert manual TKA planning methods.
• Further AI development needed for accurate angular TKA planning.

## Graphical Abstract

**Figure:**
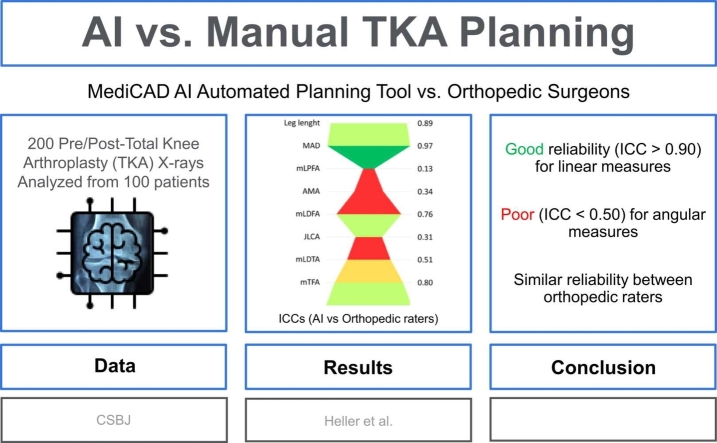

## 1 Introduction

Meticulous preoperative planning constitutes an essential component of orthopedic surgery, particularly in total knee arthroplasty (TKA) and total hip arthroplasty (THA) [1]. This planning includes the accurate assessment of the leg axis, the evaluation of potential axis deviations, and the selection of the most appropriate implant prosthesis [2], [3], [4]. It also offers numerous advantages [1]. For instance, anticipating implant component sizes can streamline hospital logistics by optimizing implant inventory and sterilization costs [5]. Furthermore, conducting systematic preoperative planning can help surgeons avoid unforeseen intraoperative challenges and has the potential to reduce operative time [6]. Ultimately, rigorous planning contributes to an optimally prepared surgical team and procedure, which in turn facilitates the best possible postoperative outcomes for each patient [1]. Achieving such preparedness necessitates a precise and critical assessment of the leg axis, potential leg-length discrepancies, and other relevant limb characteristics [2], [7]. Consequently, detailed and accurate analysis of preoperative radiographs is imperative [1]. Interactive software programs are routinely employed in clinical practice for surgical planning, enabling surgeons to perform detailed measurements of the affected limb [3]. However, this interactive approach presents limitations, such as inaccuracies in landmark placement, variability among different users, and the time commitment required for measurement execution [8], [9], [10].

In recent years, the increasing adoption of AI-assisted programs in the medical marketplace has been noted, reflecting a general trend toward progressively automated applications in healthcare [11], [12]. Potential applications span various fields, including cardiovascular and neurological imaging [13], [14], [15]. Measurement accuracy may thus be increased, and human error potentially reduced, owing to the standardized, automated procedures of AI-driven software. Significant time savings are also frequently offered by such tools [7], [8], [16]. In orthopedic radiology, these automated measurement programs demonstrate particular promise for preoperative planning, especially given the extensive mechanical considerations inherent in arthroplasty surgery [17], [18].

Numerous programs and applications currently assist in measuring lower-limb alignment angles for arthroplasty planning [3], [19]. Many of these tools function with minimal user intervention, potentially mitigating human error and variability [8], [10], [20]. Beyond primary arthroplasty, AI-assisted planning has broadened into other orthopedic applications, such as revision arthroplasty—for example, in the identification of in-situ implants in preparation for revision surgery or in the assessment of dislocation risk following THA [21], [22], [23], [24]. A more efficient preoperative planning process and improved patient care with better postoperative results are generally observed [8], [25]. Nevertheless, despite these promising developments, rigorous evaluation is required before such AI-based tools can become standard practice [26], [27], [28].

A previous study by our team evaluated the performance of an AI-supported measurement tool (LAMA, IB Lab GmbH, Vienna, Austria) for radiographic planning in TKA, highlighting both the potential and limitations of automated lower limb alignment analysis [7]. Building upon this prior experience, the present study examines the performance of the Autoplan tool in mediCAD (mediCAD Hectec GmbH, Altdorf, Germany) in comparison with manual radiographic measurements performed by two orthopedic surgeons, applying the same methodological approach. The primary objective was to assess whether Autoplan can provide accurate and consistent measurements of key lower-limb alignment parameters on pre- and postoperative radiographs in TKA patients, while also improving workflow efficiency in routine clinical practice. The Autoplan tool in mediCAD’s 2D planning software potentially offers rapid, automated landmark detection for preoperative radiographs and, consequently, swift endoprosthetic planning [29]. Underlying these tools is AI trained through techniques such as transfer learning, intersection-over-union, region-based convolutional neural networks (R-CNNs), and deep convolutional networks [30], [31], [32], [33], enabling the efficient identification of anatomic landmarks.

## 2 Material and methods

### 2.1 Study data, inclusion, and exclusion criteria

We retrospectively reviewed 200 archived radiographs (pre- and postoperative) from 100 patients (50 women, 50 men) who had undergone TKA in the past seven years at our institution (Department of Orthopedic Surgery, University of Regensburg, Germany). All included patients had appropriate radiographic imaging both before and after surgery. Radiographs were retrieved from our PACS archive, beginning with the first available scan dated January 2, 2018. Each image was reviewed for eligibility based on predefined inclusion and exclusion criteria, as well as image quality. Only cases meeting all requirements were included in the final dataset. Included were patients who met the following criteria: aged 18 years or older, underwent primary TKA for gonarthrosis within the last seven years, had standardized full-length AP standing lower-extremity radiographs both pre- and postoperatively, and had digital radiographs obtained within the last seven years. Exclusion criteria included fractures of the operative leg visible on the radiograph, postoperative radiographs showing implant failure, and preoperative radiographs demonstrating the presence of implants in the knee (e.g. previous TKA, unicondylar knee arthroplasty, high tibial osteotomy, screws, or plates for periarticular fractures), poor image quality that prevented consistent identification of key landmarks, and cases where the indication for TKA was for reasons other than gonarthrosis (e.g. trauma, neoplastic lesion). The variables assessed included leg length measured according to the Mikulicz line (from the femoral head center to the middle point of the distal tibial joint line), mechanical axis deviation (MAD), mechanical lateral proximal femoral angle (mLPFA), anatomical mechanical angle (AMA), mechanical lateral distal femoral angle (mLDFA), joint-line convergence angle (JLCA), mechanical medial proximal tibial angle (mMPTA), and mechanical tibiofemoral angle (mTFA).

We also recorded the time required for measurements by both the Autoplan tool and by manual procedure. For the Autoplan tool, measurement time was defined strictly as the automated calculation period without human interaction. For manual measurements, time started after the calibration of the radiographic image in the software and ended upon completion of all required landmarks and measurements for that image.

### 2.2 Evaluation of the radiographs (Autoplan vs. Manual)

**Fig. 1:**
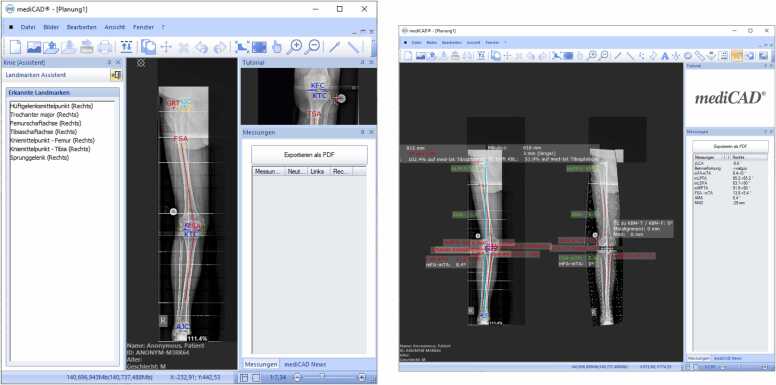
Leg alignment measurement performed by the Autoplan tool (MediCAD). The upper window (A) displays the anatomical landmarks recognized by the AI. The lower screenshot (B) presents the AI's measurement results in the summary box located on the right of the screen.

Each radiograph was analyzed using two methods: (1) the Autoplan tool by mediCAD (Version 7.0), and (2) manual measurements by two orthopedic specialists performed with the same MediCAD Software (Fig. 1). Rater A (SP) was a resident with less than 5 years of orthopedic experience, and Rater B (GM) was a senior surgeon with a decade of professional experience. Both orthopedic specialists independently assessed the same 200 radiographs, blinded to the Autoplan tool results. Rater A repeated the measurements on the same images four weeks later to assess intra-rater reliability.

Our clinic routinely includes a calibration sphere in all preoperative radiographs as a standard reference. This radiopaque marker, placed at a known distance from the X-ray source, is essential for accurately scaling the images so that real-world distances can be reliably extracted from the radiograph. However, since postoperative radiographs do not include the calibration sphere, we applied indirect calibration by referencing known dimensions from the preoperative radiograph (e.g., femoral head size or hip prosthesis head diameter). These landmarks served as surrogate reference points for the postoperative measurements.

### 2.3 Statistical analysis

Intraclass correlation coefficients (ICCs) were used to evaluate the reliability of lower-limb alignment measurements obtained by the Autoplan tool and the two independent orthopedic specialists. ICCs were calculated using a two-way mixed-effects model (single measurement, absolute agreement) and interpreted per established guidelines [34]. Inter-rater reliability was determined by comparing Autoplan tool-derived values to each orthopedic specialist’s measurements, as well as comparing the three measurers to one another. Intra-rater reliability was assessed by comparing Rater A’s two sets of measurements. Pre- and postoperative readings were analyzed separately to examine reliability differences between these conditions.

A “gold standard” reference was established by calculating the mean of three measurements: Rater A’s first and second measurements and Rater B’s single measurement. The automated measurements from the Autoplan tool were then compared to this average. The Wilcoxon Signed-Rank Test was used to compare ICC values for preoperative versus postoperative conditions. Descriptive statistics (mean, standard deviation) were calculated for all measurements.

Bland-Altman plots were used to visualize agreement for both angular measurements (AMA, JLCA) and linear parameters (MAD), plotting the mean of the two measurement methods against their difference. Limits of agreement (LoA) were defined according to established clinical thresholds: ± 2° for angular measurements and ± 5 mm for linear parameters [7], [9]. Furthermore, the measurement completion times were explicitly compared between the Autoplan tool and each manual measurement instance. Paired t-tests were employed to evaluate statistically significant differences in measurement duration between the different methods and raters.

All statistical analyses were performed in SPSS (Version 29, IBM Corp., Armonk, NY, USA). Statistical significance was set at two-sided p < 0.05. Ethical approval for this study was obtained from the University of Regensburg’s Ethics Committee (reference 24–3937–104, 29 October 2024).

## 3 Results

A total of 200 radiographic images (pre- and postoperative) from 100 patients were included in the study. Of these, proper measurement by the Autoplan tool was not feasible in three cases. In one patient case, automated measurement could not be conducted preoperatively and postoperatively because of an inlaying femoral nail. In a second case, automated measurement could not be executed postoperatively, as the femoral head was not identifiable due to excessive soft tissue density. As a result, 197 radiographs were ultimately available for performance evaluation and statistical analysis involving the Autoplan tool.

In ten additional cases, preoperative implanted foreign material was present. Eight cases involved an ipsilateral inlying endoprosthesis following THA, one case involved foreign material in the osseous pelvis, and one case involved a plate and cerclage in the proximal femur region together to an inlying hip endoprosthesis. However, these instances did not impact the practicability of the measurements; the Autoplan tool could still perform the measurements.

In three cases, the reference sphere could not be detected preoperatively by the software. Manual scaling of the sphere was then performed, allowing measurement to proceed automatically as intended. As the reference sphere was used solely preoperatively, this issue occurred exclusively in preoperative radiographs.

In two cases, a reference sphere was detected that was not present in the postoperative radiograph. The software then incorrectly identified certain osseous structures as scaling spheres. However, because postoperative measurement scaling was performed using the femur and its preoperative measurements as reference, this issue was not a significant concern.

ICCs demonstrated overall high reliability for leg length, MAD, mLDFA and mTFA in both pre- and postoperative conditions (Table 1). Notably, MAD exhibited excellent agreement (ICC > 0.90) between the Autoplan tool and both orthopedic specialists, indicating strong consistency for this parameter (Table 2). Conversely, mLPFA, JLCA, AMA exhibited poor reliability (ICC < 0.50) across all comparisons.

**Table 1:** Intraclass correlation coefficients (ICC) for evaluating the reliability of measurements across raters. The table includes ICC values with 95 % confidence intervals, comparing the Autoplan tool with both physician raters, inter-rater reliability between both physician raters, and intra-rater reliability for repeated measurements by one rater. The total number of measurements is reported, combining preoperative and postoperative cases. Lower ICCs observed for angular parameters such as mLPFA, JLCA, and AMA reflect known variability in landmark-based measurement and interpretation [7].

**Table 2:** ICC values comparing measurements from the Autoplan tool with the gold standard derived from the mean values of orthopedic raters. Preoperative and postoperative measurements are analyzed separately. Clinically relevant thresholds (CID, ± 2° for angles and ± 5 mm for lengths) are applied to assess agreement and highlight key discrepancies across parameters.

Comparisons between preoperative and postoperative measurements revealed generally somewhat lower ICCs following surgery for certain parameters, such as mLDFA and mMPTA (Table 2). However, the Wilcoxon Signed-Rank Test indicated no statistically significant differences in overall ICC values between pre- and postoperative conditions for Leg Length, MAD, and mTFA (p > 0.05).

**Fig. 2:**
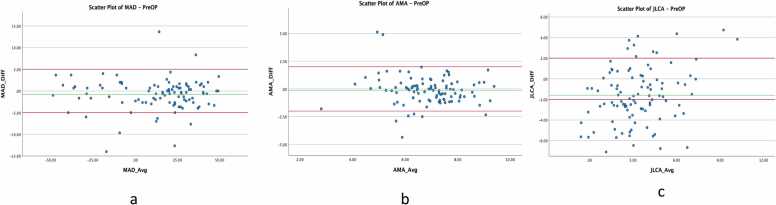
Fig. 2 - Bland-Altman scatter plots for preoperative measurements of MAD (a), AMA (b), and JLCA (c). The Y-axis represents the differences between the Autoplan tool and the mean of all rater measurements, while the X-axis shows the averages of these measurements. The green line represents the mean difference, which is close to zero for most parameters, indicating minimal systematic bias. The red lines denote clinically relevant limits of agreement ( ± 2° for angles and ± 5 mm for lengths). Substantial disagreement is observed for JLCA, with many values exceeding the acceptable range.

Bland-Altman plots (Fig. 2) further illustrate these findings. MAD measurements clustered tightly around the zero-difference line, while AMA and JLCA showed greater scatter, particularly at higher angle values (Figs. 2B and 2C).

**Fig. 3:**
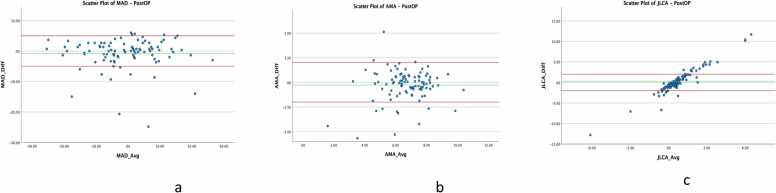
Bland-Altman scatter plots for postoperative measurements of MAD (a), AMA (b), and JLCA (c). Agreement improves significantly for JLCA compared to preoperative measurements, with fewer outliers and reduced bias. However, a proportional trend suggests variability at higher values, likely due to measurement errors in the Autoplan tool.

Postoperative Bland-Altman plots (Fig. 3) revealed similar patterns. MAD demonstrated high agreement, with most data points falling within the limits of agreement ( ± 5 mm), confirming strong postoperative reliability (Fig. 3 A). In contrast, angular measurements like AMA and JLCA continued to show noticeable variability, especially at higher values (Figs. 3B and 3 C). Although variability decreased slightly for JLCA postoperatively, proportional biases remained evident, indicating discrepancies increased with larger measurements.

Marked differences were observed in the time required for measurement completion between manual measurements and the automated measurement by the Autoplan tool. The Autoplan tool required a significantly shorter duration for completion (mean: 2.4 seconds, SD: 1.6 seconds) compared to manual measurements by either orthopedic specialist (mean range: 43.2 – 48.3 seconds, p < 0.001) (Table 3).

**Table 3:** Overview of all preoperative and postoperative measurements conducted by the Autoplan tool, the less experienced rater (A) who repeated the same measurements (1^st^) after four weeks (2^nd^), and the more experienced rater (B). Mean and standard deviation values are presented for each parameter to summarize the variability across raters and measures.

	**Autoplan (n = 197)**		**Rater A 1**^**st**^**(n = 200)**		**Rater A 2**^**nd**^**(n = 200)**		**Rater B (n = 200)**	
	Mean	SD	Mean	SD	Mean	SD	Mean	SD
**Leg length (mm)**	790.6	54.8	792.5	53.8	789.3	53.9	792.0	60.6
**MAD (mm)**	5.8	20.1	6.6	20.2	6.6	20.0	6.5	20.7
**mLPFA (°)**	95.1	18.8	93.7	22.3	90.9	5.8	90.4	6.6
**AMA (°)**	6.7	1.5	6.8	2.6	7.2	2.0	6.7	1.1
**mLDFA (°)**	89.0	3.5	88.9	2.6	89.1	2.6	89.1	3.1
**JLCA (°)**	1.8	3.0	2.3	2.3	2.8	6.2	2.6	2.6
**mMPTA (°)**	89.2	4.3	88.8	3.2	88.2	6.0	88.6	3.1
**mTFA (°)**	−1.7	6.0	−1.7	6.0	−1.6	8.6	−1.9	5.9
**Time (s)**	2.4	1.6	48.3	6.6	44.9	7.4	43.2	4.2

n: total measurements, SD: Standard Deviation

Additional descriptive statistics, including variability in linear and angular measurements, are presented in Table 3.

## 4 Discussion

The findings of the present study indicate that the Autoplan tool provides notable advantages; however, limitations exist in the accuracy of certain measurements when compared to specialist assessments. Our results demonstrated high reliability and efficiency for linear parameters, such as MAD and leg length. Given these advantages, including the significant reduction in time required, automated measurement by the Autoplan tool clearly offers an advantage over conventional manual measurements for linear parameters. Interestingly, the poor reliability of mLPFA, JLCA, AMA (ICC < 0.50) was not limited to the comparison between the Autoplan tool and manual measurements, but was also apparent among the two experienced orthopedic specialists. This suggests that the variability may stem from the inherent limitations in radiographic landmark visibility, particularly postoperatively where implants can obscure reference structures. Previous work has shown that even under standardized imaging conditions and across different levels of clinical experience, alignment measurements are subject to altered anatomy, difficulty in defining midpoints on prosthetic surfaces, or subtle changes in limb positioning [43]. Such variability likely contributes to the reduced agreement observed in our study and underscores the need for enhanced standardization in both imaging protocols and measurement algorithms [3].

Similar to our findings with the Autoplan tool, Hoffmann et al., who also evaluated this software, reported excellent accuracy for leg length (ICC > 0.99) and overall alignment (ICC > 0.97), but encountered difficulties with joint-level angles, particularly in postoperative measurements [9]. It should be noted, however, that their ICCs were calculated by comparing AI measurements to the average of two observers, whereas our analysis compared AI directly to each rater individually. This may partly account for the slightly higher ICC values reported in their study.

Our team's previous investigation by Pagano et al. evaluated another AI-supported automated measurement program (LAMA), which demonstrated good to excellent agreement for several parameters (e.g., MAD with ICC = 0.98), but showed lower ICC values for angular measurements and particularly struggled with postoperative radiographs and in obese patients. Building on that work, the present study utilized the same radiographic dataset to compare the performance of the mediCAD Autoplan tool under identical imaging and statistical conditions. While both AI tools encountered challenges with angular parameters such as JLCA and AMA, the Autoplan tool exhibited improved measurement feasibility, with a lower failure rate and more consistent landmark detection across pre- and postoperative images (Table 4). These differences underscore the variability in performance among AI-based tools and the need for independent validation of each system before integration into clinical workflows [7].

**Table 4:** Intraclass correlation coefficients (ICCs) reported in the present study, Pagano et al. [7], and Hoffmann et al.[9] for evaluating the agreement between AI-based software (Autoplan from MediCAD and LAMA) and human raters in radiographic planning for TKA. Preoperative and postoperative values are reported separately when available. Sample sizes represent the number of radiographs or limbs analyzed per study. Across all studies, ICCs were consistently high for global alignment parameters such as mechanical tibiofemoral angle (mTFA) and leg length, while joint-level angular parameters (e.g., JLCA, mLDFA, mMPTA) showed lower agreement.

Substantial differences were observed in the time required for measurement completion between manual measurements and automated results from the Autoplan tool. The Autoplan tool operated considerably faster throughout the study. While a trend of reduced time expenditure was observed for the orthopedic specialists due to a learning effect, the Autoplan tool presented a substantial advantage in terms of efficiency.

These results provide valuable insights into the potential benefits of integrating automated AI-driven measurement in routine clinical practice. With few exceptions, the Autoplan tool functioned effectively for most examined radiographs, both pre- and postoperatively, showing strong agreement with the results obtained by the orthopedic specialists, particularly for linear parameters. Through automated planning, some potential sources of human error and investigator-dependent deviations could be reduced, and measurements could be performed more uniformly. In this way, a substantial increase in efficiency may be achieved, making the clinical workflow to become more standardized and faster, especially in a high-volume clinical setting.

In their studies, other authors drew similar conclusions, suggesting that automation of measurements, reduction of inter- and intra-observer variability, and time savings could be achieved through the integration of AI-based automated planning in clinical workflow [10], [35], [36]. For instance, Schock et al. [10] demonstrated significant time efficiency gained through automated analysis of lower limb alignment, a finding directly mirroring our observation. Furthermore, Bonnin et al. [35] provided evidence for the potential of AI to enhance standardization and reduce interobserver variability in radiographic assessments. Lambrechts et al. [36], also in the context of total knee arthroplasty planning, found that AI-generated plans were comparable to surgeon-approved plans, further supporting the idea that automation can streamline workflows without necessarily sacrificing accuracy, particularly in certain aspects of planning.

However, limitations and errors in the autonomous application of AI were also evident during the automated measurement by the Autoplan tool. The observed poor reliability of angular measurements, which was also present in our manual assessments, reflects the difficulty of consistently defining landmarks in complex anatomy or when interfering factors are present. Specific factors that interfered with the software, resulted in either erroneous results or an inability to perform measurements. For example, patient-specific factors, such as the presence of foreign material (e.g., an inlaying femoral nail) or obesity, could impede automated measurement by preventing accurate recognition of the femoral head as a reference point. Consistent with findings from prior investigations of other AI-based measurement software [7], [9], the present study also encountered challenges in automatically measuring certain radiographs due to image complexities and observed lower reliability in angular measurements compared to linear measurements. The AI, in its current form, seems to reflect the inherent challenges that also exist in manual radiographic assessment of these specific angles. It is possible that the AI is consistently identifying and measuring landmarks, but the underlying variability in manual landmark identification contributes to the low agreement. This finding also raises questions about the 'gold standard' itself, as significant variability even amongst experienced raters suggests that a single 'true' value for these angular measurements might be difficult to ascertain from radiographs alone [44].

A potential explanation for the challenges in angular measurements lies in certain limitations of current algorithms. As highlighted by Lambrechts et al. [37], manufacturers’ default plans often necessitate significant surgeon modification. Their work demonstrates machine learning's potential for generating more surgeon- and patient-specific plans, thus reducing subsequent need for corrections.

Several limitations of this study warrant consideration when interpreting the results. The automated assessment depends on the image quality of the radiographs. Various confounding factors can influence the automated measurement process, potentially leading to inaccurate results. These factors include image quality, contrast reduction due to soft tissue shadows, the method of image acquisition (e.g., rotation of the legs, affecting the position of landmarks such as the greater trochanter or center of the knee), or incomplete image capture (e.g., showing only a portion of the femoral head). When interpreting the results, particularly concerning their potential future application in clinical practice, the possibility of bias arising from the authors' evaluation of new approaches compared to established standards should be acknowledged. Therefore, the available results limit the ability to draw broad conclusions about the general use of AI in preoperative planning. While the study included a postoperative scaling method based on anatomical references (e.g., femoral head diameter), the accuracy and repeatability of this indirect approach were not formally evaluated. We acknowledge that this may introduce variability in postoperative linear measurements. However, as the same anatomical reference was used consistently across both manual and automated measurements for each case, the relative comparison remains valid.

Further research is required to establish standardized usage of automated measurement tools in future routine clinical practice. Initially, the algorithms underlying these tools require further refinement through machine learning to better handle angle measurements and adapt to diverse anatomical conditions, as this was the most challenging aspect identified in our findings. This refinement can be achieved through further validation by collecting larger, diverse datasets, and conducting real-world clinical studies to assess generalizability and utility [37], [38], [39]. To enhance the accuracy and reliability of automated measurements, using complementary AI-based tools, where appropriate, seems advisable for a more comprehensive approach to preoperative planning [40].

## 5 Conclusion

While some caution against exclusive reliance on fully automatic algorithms due to accuracy concerns compared to manual measurements [41], [42], our findings also highlight the limitations and inherent variability within manual measurements, particularly for angular parameters. The Autoplan tool demonstrates clear benefits in efficiency and reliability for linear measurements, but further development is needed to improve angular assessments, both for automated tools and to address the challenges in achieving consistent manual angular measurements.

## CRediT authorship contribution statement

**Renkawitz Tobias:** Supervision. **Pagano Stefano:** Writing – review & editing, Validation, Methodology, Formal analysis, Conceptualization. **Schuster Marie Farina:** Investigation, Data curation. **Forchhammer Lina:** Investigation, Data curation. **Scharf Markus:** Investigation, Data curation. **Heller Marie:** Writing – original draft, Validation, Methodology, Investigation, Formal analysis, Data curation. **Maderbacher Guenther:** Formal analysis, Data curation.

## Declaration of Generative AI and AI-assisted technologies in the writing process

During the preparation of this work the authors used Gemini Version 2.0 from Google AI Studio exclusively for grammar correction and text style refinement of the original manuscript. After using this tool, the authors reviewed and edited the content as needed and take full responsibility for the content of the publication.

## Declaration of Competing Interest

Tobias Renkawitz declares the following financial interests: Research funding at personal disposal from DePuy, Zimmer, Aesculap, German Federal Ministry of Education and Research, Deutsche Arthrose-Hilfe, OttoBock-Stiftung, German Federal Ministry of Economic and Development, Oskar-Helene-Heim Foundation in Berlin, Vielberth Foundation, and Deutsche Forschungsgemeinschaft (DFG). He has received reimbursement of costs from DePuy, Zimmer, Aesculap, Federal Ministry of Education and Research, Deutsche Arthrose-Hilfe, OttoBock-Stiftung, Federal Ministry for Economic Cooperation and Development, Oskar-Helene-Heim Foundation in Berlin, Vielberth Foundation, DGOOC, BVOU, and DGOU. He has also received reimbursement of costs for training/lectures from DePuy, Zimmer, Aesculap, German Society for Endoprosthetics (AE), and Bavarian Association of General Practitioners. His non-financial interests include his position as Chair (W3) of Orthopedic Surgery, University of Regensburg, and Director of the Orthopedic Department for the University Hospital Regensburg. He holds memberships as Vice President of the Professional Association for Orthopedics and Trauma Surgery (BVOU), in the General Board of the DGOOC, as Head of the “Arbeitsgemeinschaft Evidenzbasierte Medizin” of the DGOU, on the editorial board of “Die Orthopädie” and “Die Unfallchirurgie” (SpringerMedicine), as editor of the “Orthopädie und Unfallchirurgie—Mitteilungen und Nachrichten” (OUMN), and on the International Advisory Board of the Journal of the American Academy of Orthopedic Surgeons (AAOS).
